# A Recommendation System Based on AI for Storing Block Data in the Electronic Health Repository

**DOI:** 10.3389/fpubh.2021.831404

**Published:** 2022-01-21

**Authors:** Vinodhini Mani, C. Kavitha, Shahab S. Band, Amir Mosavi, Paul Hollins, Selvashankar Palanisamy

**Affiliations:** ^1^Department of Computer Science and Engineering, School of Computing, Sathyabama Institute of Science and Technology, Chennai, India; ^2^Future Technology Research Center, College of Future, National Yunlin University of Science and Technology, Yunlin, Taiwan; ^3^Faculty of Civil Engineering, TU-Dresden, Dresden, Germany; ^4^Institute of Information Society, University of Public Service, Budapest, Hungary; ^5^John von Neumann Faculty of Informatics, Obuda University, Budapest, Hungary; ^6^Cultural Research Development School of Arts, Institute of Management, University of Bolton, Bolton, United Kingdom; ^7^Intelligent Automation, Ford Motors Pvt. Ltd., Chennai, India

**Keywords:** artificial intelligence, machine learning, health repository, patients, health data, storage, deep learning

## Abstract

The proliferation of wearable sensors that record physiological signals has resulted in an exponential growth of data on digital health. To select the appropriate repository for the increasing amount of collected data, intelligent procedures are becoming increasingly necessary. However, allocating storage space is a nuanced process. Generally, patients have some input in choosing which repository to use, although they are not always responsible for this decision. Patients are likely to have idiosyncratic storage preferences based on their unique circumstances. The purpose of the current study is to develop a new predictive model of health data storage to meet the needs of patients while ensuring rapid storage decisions, even when data is streaming from wearable devices. To create the machine learning classifier, we used a training set synthesized from small samples of experts who exhibited correlations between health data and storage features. The results confirm the validity of the machine learning methodology.

## Introduction

In the modern era, clinicians no longer manage health data exclusively, but are increasingly responsible for obtaining consent from patients (1). The rights of patient's access to, analysis of, and exchange of their health information have evolved dramatically (2). The majority of patients are dissatisfied with their health care providers after sharing self-tracking data (3). It is still possible to enhance patient health care by incorporating patient health data into the current health data systems. Literature has identified various categories of patient health information (4). These categories include information about medications, biometrics, behavioral information, data about social interactions, genetics, psychological data, data about symptoms, and reports. Blockchain-based interplanetary file system secondary storage of health data has been implemented to safeguard the privacy and security of patient health information (5). Yet very few studies have evaluated how patients' health data is stored. A key component of the proper management of health data is protecting the privacy and confidentiality of the patient while maintaining data accessibility for relevant stakeholders. Studies indicate that health data security poses a massive threat. This is evidenced by the proliferation of medical devices with limited memory and power (6, 7) and substantial medical data repositories (8). Many types of organizations are responsible for managing the massive amount of health data.

Health data is often portrayed as being sensitive to all patients with the same level of privacy and confidentiality; however, this is not true in practice because it is not equally sensitive to everyone at the same time. When a patient reaches a high level of public prominence, she may surrender the ECG data she generated on her own and to her cardiologist. This data can be accessed by other healthcare providers through an electronic health record. A patient who wishes to keep her pregnancy test results private may be forced to allow her provider to store her pregnancy test results. The dissemination of health data between multiple providers who manage data repositories now enables the storage medium to be customized based on patient needs. This includes the cost, size, security, confidentiality, and privacy of each chunk of data. Hybrid execution models, such as those described by the author (9), allow sensitive data to be stored in private clouds while no sensitive data is maintained in public clouds. Nevertheless, it does not specifically address health data processing. Communication between the two cloud platforms also takes time, and computations that rely on bandwidth use a lot of resources. A hybrid cloud platform was developed by (10) for solving this problem. Medical sensors, apps, and devices provide data to artificial intelligence, which enables the automatic diagnosis of health conditions. Health data, including ECG, blood pressure, and pulse rate, can be classified as normal or abnormal by algorithms based on a range of conditions and thresholds set by healthcare professionals. Clinical research and clinical care are usually aided by abnormal data. Using the Body Area Sensor Network, (8) developed an agent-based system developed for elderly people to preserve abnormal data. Health information is generated in enormous quantities nowadays, so a diverse storage solution is needed (11). Several researchers have examined the performance and cost parameters of various Cloud Service Providers (CSPs) to design methods for selecting suitable CSPs for storing consumers' data (12–14). High-performance cloud services minimize the time spent in operations but incur high costs. Additionally, researchers are investigating blockchain technology for its promise of security and privacy for health data management. Combining blockchain-based eHealth with traditional health databases is possible, which can be arranged based on users' preferences and the possibility of utilizing the data in the future. However, due to the design of blockchains, they are not suitable for hosting large amounts of health data. A software agent that knows the patient's preferences is inserted inside the application in (15). Nonetheless, they never described a way to make this decision. To assist in choosing storage repositories, we developed a model that incorporated not only (8)'s criteria, but also aspects like data confidentiality, privacy, and quality of performance.

### Motivation

Every Blockchain miner owns a local ledger, so this technology allows transactions to be verified and processed without the need for third parties. Verifying transactions does not require a centralized server. Document alterations cannot be guaranteed through conventional database storage and blockchain-based hash management. Data is only detectable in a blockchain if a hash pointer holds a pointer to it. Depending on the patient, personal preferences, and other factors, the sensitivity and significance of the health information are also different from repository to repository. Choosing the right repository is extremely crucial. As wearable sensors continuously stream health data, the challenges are exacerbated. In (16), the author has surveyed the importance of artificial intelligence in healthcare. The prediction of COVID-19 infected patients using artificial intelligence has been implemented in (17), but there is a need for an appropriate repository to store the data.

### Contribution

**Figure 1:**
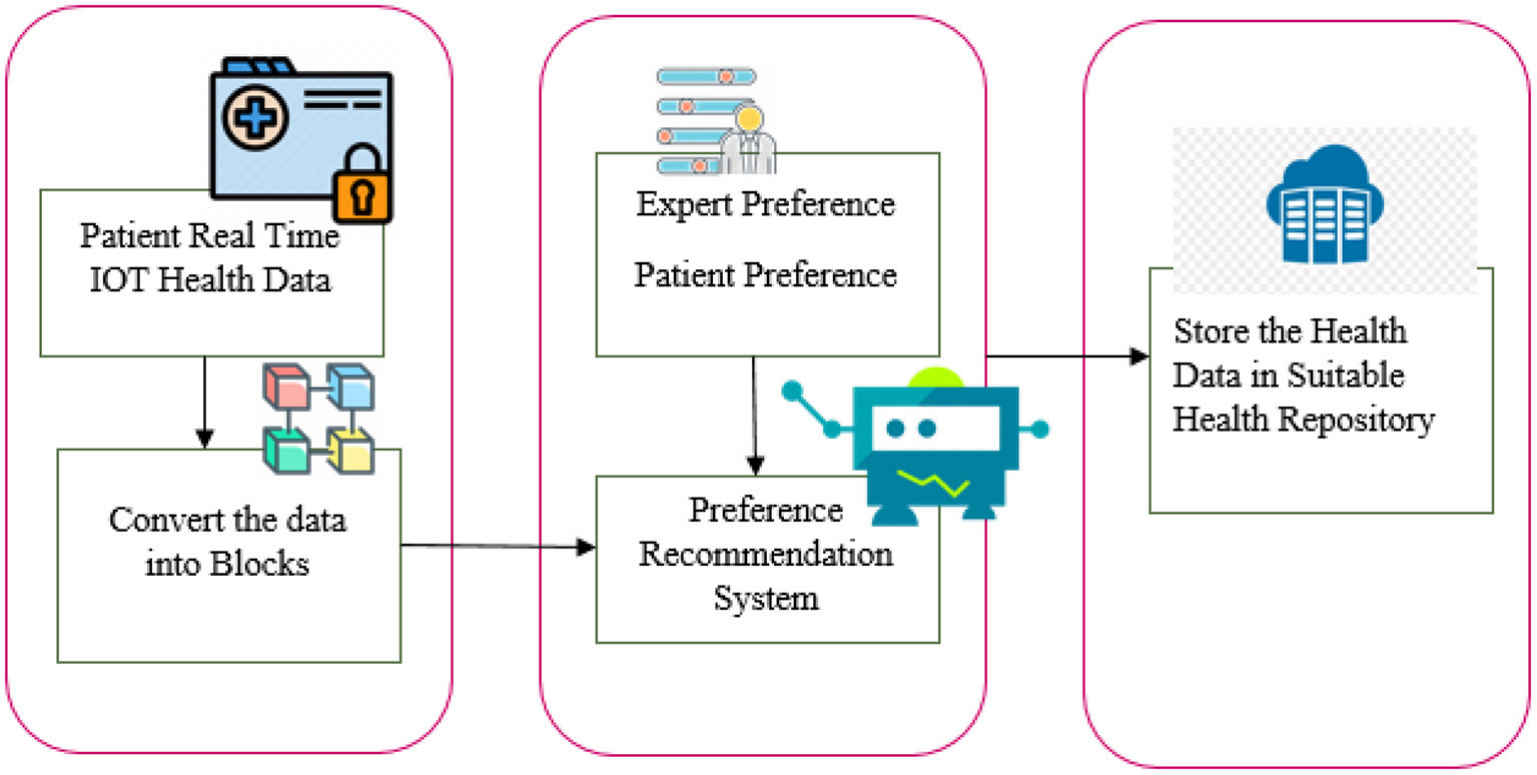
Paper contribution flow diagram.

In our research, we considered the variation in data sensitivity, volume, and other factors to locate the appropriate system to manage health records. The flow diagram of the paper contribution is shown in Figure 1. Collect the health data and health repository parameters. Evaluations of both health information and health repository parameters are given a score. The machine learning-based recommendation model for health data storage proposes a way to distribute health data among multiple repositories. A model for automated health data storage recommendation is being developed to determine appropriate storage repositories. Through correlation analysis, user preferences, and clinical heuristics, a machine learning-based classifier is used to map health data characteristics to each repository. Patients' security and privacy preferences are taken into account as well as the sensitivity of health data.

### Organization

Following are the sections of the paper: section Background addresses related work. In section Model for Recommendation of Health Repositories, we present the proposal for a recommendation model for a health repository. Section Implementation describes how the system will be implemented. The results and evaluation of performance will be discussed in section Results and Discussion. Conclusions and future work will be discussed in section Conclusion.

## Background

Big Data cannot be stored, accessed, or analyzed with a single health record system. Patients can lose medical information when their electronic health records are malfunctioning (18). Due to the manual uploading of data generated by wearable sensors to personal health records, caregiver responses were delayed. For this reason, (19) developed methods for storing patient-generated health information on commercial blood glucose monitors. The electronic health record system could be made to fit the streamed data if it is filtered or compressed (20). In (21–24), a number of action plans and standards were advocated for the adoption of an electronic health record system. A selection of an electronic health record should take into account functional requirements, troubleshooting, and optimization features (22). The author provides a list of steps to follow before buying an electronic health record system. Checklists mostly cover client meetings on site, site visits, and maintaining live workflows. Health data sources such as hospitals, clinics, insurers, and patients should be integrated into centralized databases, according to the author (25). In particular, patient-centered health data with high degrees of structural heterogeneity must be stored and processed quickly because of their high volume and rate. For health data, to provide useful insights, precision is essential, but some sources produce vague and inaccurate information. Distributed data storage systems do offer some relief to these issues (26). Various cloud storage mediums have been examined. A machine learning and deep learning model is used to predict the thermal sensation vote system (27). Utilization of a compression algorithm to retrieve the health repository data as fast as possible using blockchain and interplanetary file systems (IPFS) without data loss (28). Diabetic Retinopathy is efficiently classified using a deep learning and machine learning algorithm (29). Genetic algorithm with fuzzy logic is a tool to help medical practitioners diagnose heart disease at an early stage using adaptive genetic algorithm with fuzzy logic (AGAFL) (30). Health data storage systems and data properties were not considered in the selection of repositories. Furthermore, no machine learning mechanisms were developed to cater to user preferences.

In the next section, we describe how we facilitate distributed health data management.

## Model for Recommendation of Health Repositories

As data streams increase, the need for storage decisions becomes more frequent, making manual consultation with patients an inefficient process that requires an automated solution. It is, however, impossible to prespecify the data storage requirements for each patient that will apply to all possible future contexts. The learning classifier may generalize to a broader range of mappings based on a manual mapping specification by an expert.

**Figure 2:**
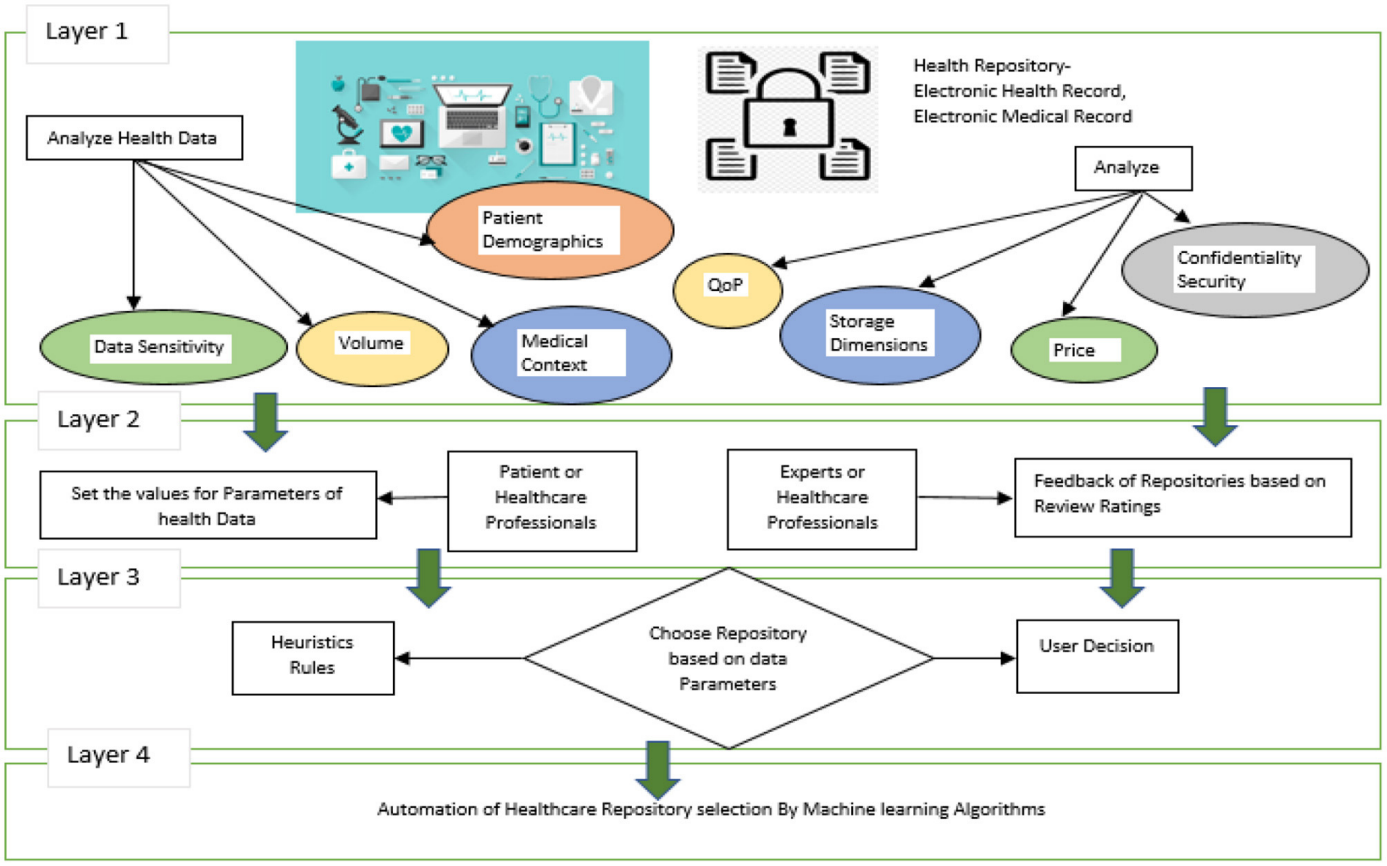
Proposed system architecture.

**Figure 3:**
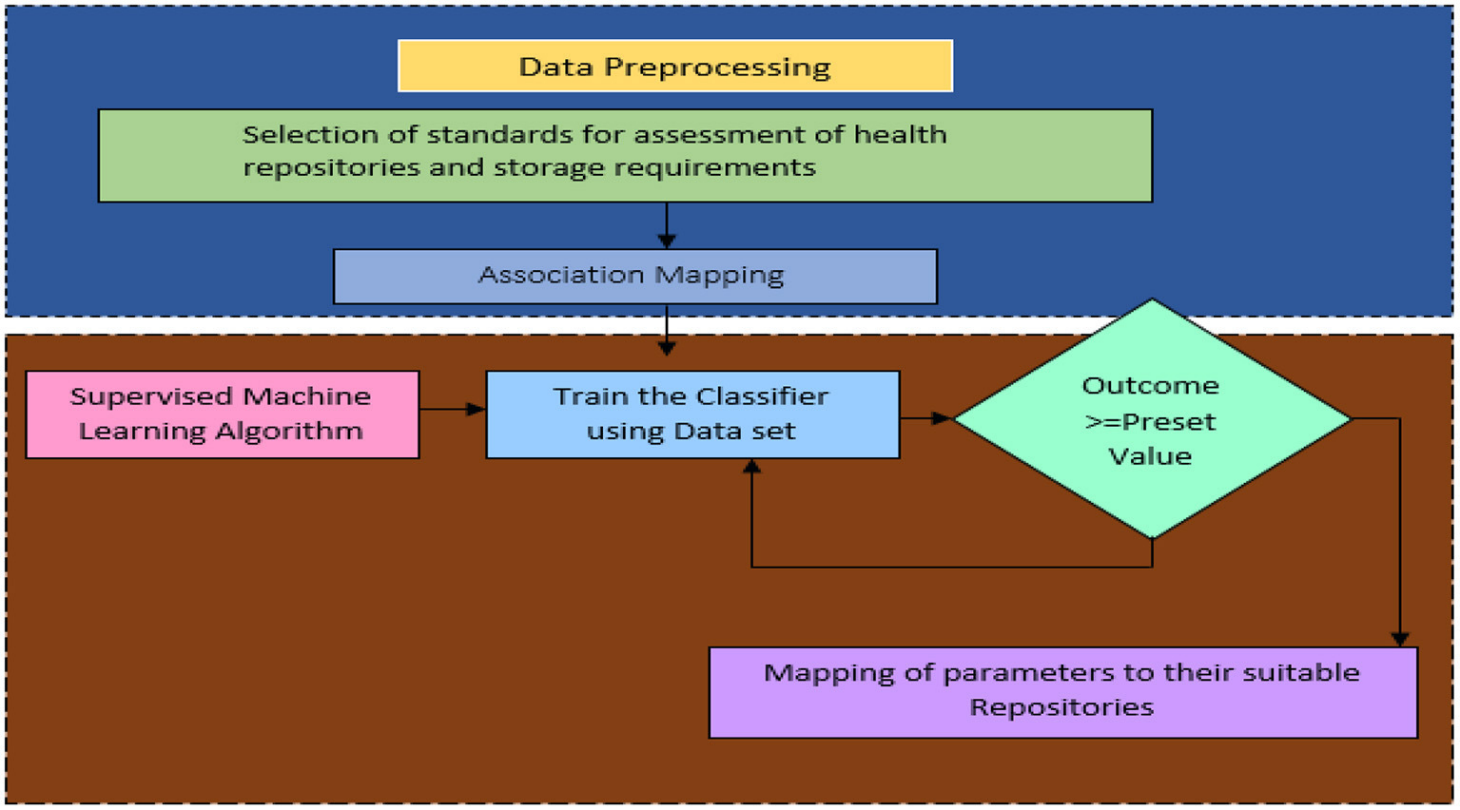
Proposed health repository recommendation system.

The following sections explain in detail the overall approach described in Figures 2, 3. Data storage requirements - an illustration of which is displayed in layer 1 of Figure 2, consists of a set of variables or features that characterize the requirements for storing a chunk of data. Some of the attributes' values have been shown to be numerical [1–10] and others to be qualitative. Secondly, each instance of the dataset contains the specifications required to store each chunk of data as shown in Figure 2.

Health Repository Evaluation Criteria are calculated in layer 3 by adding a rating provided by an expert group. These criteria reflect the characteristics of storage repositories as shown in Figure 2. Three standards apply to rank five storage repositories. Medical professionals and patients themselves may create clinical heuristic rules in layer-3 of Figure 2 and each instance in the dataset is categorized according to the preferences of the users. A storage repository can be assigned to an instance based on heuristic rules in a real-world situation. The correlation coefficient offers an inference of a class label when preferences and heuristics do not match well. The health repository requirements can be mapped to layer-4 (user and expert expectations) by a machine learning classifier, as shown in Figure 2. In Figure 3, a recommendation framework for health repositories is illustrated. There are two parts to the framework: determining which standards should be used for the storage and assessment of data and implementing machine learning.

## Implementation

This recommendation system assumes that a patient is in full control of his or her decision regarding storage. It is impossible to make decisions manually in many cases because they are made so frequently. Hence, automated processes are essential. In the mapping process, the characteristics of a repository managed by an agent group are matched with the characteristics of data about the storage requirements of patients. Because patients' storage requirements vary so much, it is impossible to predetermine every possible scenario. By utilizing a set of mappings that is specified manually by experts, machine learning is used to generalize a mapping over a wide range of patient contexts. This methodology involves defining a set of attributes that describe what chunk of data needs to be stored. There are numerical values and categorical values assigned to those attributes. Thus, a dataset containing these attributes will be created, with each instance representing a different set of storage requirements. A group of experts' ratings are then used to determine the characteristics of the available storage mediums. To determine what class each instance falls into, statistical correlation and heuristic rules are employed. Based on the training datasets, the supervised machine learning classifier maps the data into a storage repository. Figure 3 illustrates two components of the recommendation system: Data Pre-processing and Supervised Machine Learning. According to Figure 3, the upper portion of the framework contains the characteristics of the data storage requirements. There are a number of features that demonstrate the characteristics of health repositories. A number of associations were found between the two groups of features.

### Data Preprocessing

The data collected from hospitals and patients undergoes a preprocessing process, which includes analyzing data storage requirements, identifying sensitive data areas, analyzing the volume of each record, analyzing the patient health profile, determining the demographics of patients, and analyzing health repository parameters as well as storage, cost, security, privacy, and performance.

#### Characteristics of Data Storage Requirements

To determine which repository is the best option, consideration is given to the sensitivity of the data, the volume of the data, medical care context, and demographics of the patient.

##### Sensitivity of the Data

It is imperative to prevent unauthorized access to all health-related data. Depending on the data type, some breaches are more likely than others. Depending on the individual's preferences and context, the level of data sensitivity may vary.

##### The Volume of the Data

Reports, medical diagnoses, and medication summaries are not frequently created, which means that their storage needs are less than those of health data sets.

##### Context of Medical Care

The context may be palliative care, critical care, chronic illness, or no chronic illness. The context may also differ based on the country.

##### Demographics of Patients

Several factors can play a significant role in determining which storage medium to use, such as socioeconomic status, occupation, education, and nationality.

#### Health Repository Evaluation Parameters

Evaluation parameters for health repository such as security, privacy, cost, storage capacity, and performance. Table 1 shows the parameters and criteria of the health repository evaluation.

**Table 1:** Health repository evaluation.

**Assessment parameters**	**Survey questions for health repository ratings**
Storage	Can the repository be used to store Big Data?
	Regarding processing Big Data, what is the repository's role?
	Are there any benefits to storing continuously streamed data in the repository?
Cost	Does deployment cost a lot?
	Does maintenance cost much?
	What is the service cost?
Security	Is the storage repository capable of maintaining data integrity?
	Does the storage repository have 24/7 accessibility?
	Are storage repositories resistant to cyberattacks?
Privacy	Is data accessible to third parties?
	Is the access control right given to the owner of the health records?
Performance	How fast can you upload files?
	Is it possible to retrieve data quickly?
	Is it possible to process data quickly?

#### The Relationship Between Repository Evaluation Standards and Data Features

Medical records, in particular those generated by patients, are to be transferred to a health record system that reflects the preferences of the user and the data requirements. Health data requirements and criteria for evaluating storage are correlated in a one-to–to-many fashion as implemented in Algorithm 1. Some associations are strong, and some are weak. To facilitate the rapid processing of highly confidential data, a health record system may accept data blocks in plaintext format. Data with relatively low confidentiality can be highly sensitive due to the demographic characteristics of patients. Data about a patient's demographics, such as their educational background and professional experience, may affect their privacy concerns. Users can then choose from a variety of storage repositories that protect their confidentiality. The sample association mapping as shown in Table 2.

**Algorithm 1:** Association mapping ().

Step 1: Begin
Step 2: Let Data Source as DS;
Step 3: Let Storage Requirements as SR;
Step 4: Let Health Repository Parameters as HRP;
Step 5: For each data ϵ DS do
Step 6: For each Storage Requirement ϵ SR do
Step 7: Collect the data;
Step 8: Identify the SR;
Step 9: Collect the HRP;
Step 10: For each SR and HRP do
Step 11: Analyze the parameters using Evaluation
Criteria;
Step 12: If (SR ϵ HRP)
Step 13: SR (SR1…n) → HRP (HRP1…n);
Step 14: Create Association Dataset as AD;
Step 15: Else
Step 16: Print Not Associated;
Step 17: End; End; End; End; End;

**Table 2:** Association mapping.

**S. No**	**Characteristics of data storage requirements**	**S. No**	**Health repository evaluation parameters**	**Association mapping**
1	Sensitivity of the data	A	Storage	1 → (B,C,D,E)
2	The volume of the data	B	Cost	2 → (A)
3	Context of Medical Care	C	Security	3 → (E)
4	Demographics of patients	D	Privacy	4 → (B,C,D,E)
		E	Performance	

### Supervised Machine Learning Algorithm

Dynamically suggest health repositories based on supervised learning for particular data blocks, which is implemented using Algorithm 2. A training dataset must be generated for every instance of the dataset in addition to the labeled training datasets. Health repositories will be assigned data blocks that have a number of attributes. Among the attributes are some that are directly linked to the data block and others that are directly linked to the patient. Attributes include data sensitivity, volume, context of care, and demographics of the patients. The health repository should consider for evaluation such as electronic health records, cloud based electronic health records, blockchain based electronic health records, patient health record, and Electronic Medical Records. We considered the following health repository parameters in this study: security, privacy, cost, storage capacity, and performance. Each repository has been assigned a rating value ranging from 1 to 10.Whenever other attributes are not significant in determining the health repository, a linear regression Y (1) is calculated to label the instance as shown in Equation 1.


(1) $$\begin{array}{l} Y=A+RX \end{array}$$

(2) $$\begin{array}{l} \text{R}\;=\;\text{n}\left(\overset{\text{n}}{\underset{\text{i}\;=\;1}{\sum }}\text{xiyi}-\left(\overset{\text{n}}{\underset{\text{i}\;=\;1}{\sum }}\text{xi}\right)\left(\overset{\text{n}}{\underset{\text{i}=1}{\sum }}\text{yi}\right)\right) \end{array}$$

(3) $$\begin{array}{l} \text{A}=\;\frac{\left(\sum_{\text{i}\;=\;1}^{\text{n}}\text{yi}\right)-\text{R}\left(\sum_{\text{i}\;=\;1}^{\text{n}}\text{xi}\right)}{\text{n}} \end{array}$$

Where R is the Coefficient which contains R1,R2,R3,R4,R5,R6,R7,R8,R9,R10 are calculated between the set of data storage requirements(DR) as shown in equation 2. Here are the evaluation criteria for Electronic health record (D1), Patient health record (D2), Cloud-based electronic health record (D3), Blockchain-based electronic health records (D4), and Electronic Medical records (D5). The calculation of health repository recommendation Di is estimated using the equation:


(4) $$\begin{array}{l} \text{Di}=\text{High}(\text{R}1,\text{R}2\ldots ..\text{Rm}) \end{array}$$

M is the number of health repositories and n is the rating criteria. Secondly, the choice of a health data repository can be influenced by the decision of the healthcare professional, the preferences of the user, and a variety of factors such as normal or abnormal behavior patterns and patient health status, as well as other demographic factors. Patients with unusual health patterns should store their health records in a repository that health care professionals can access quickly. A less secured and less expensive repository can be used to store data which is hardly ever accessed by health care professionals. Different users may have different privacy preferences, and those preferences may change over time based on different contexts (31). The health record system for a patient should take into account a variety of factors. There are several factors involved, such as medical conditions, personal characteristics, socioeconomic status, as well as the type and significance of data. The level of privacy and security preferences of individuals may change over time as well. In contrast to patients with terminal illnesses, young individuals may be more concerned with privacy and security. By considering author preference, some of the sample user preference and health professional preference heuristic rules were implemented, as shown below:

1. If (Data= standard && volume=large)
  Then
  Storage Repository=Cloud based Health Record Management System
2. If (Data= standard && volume=low)
  Then
  Storage Repository=Blockchain enabled Personal Health Record System
3. If (Data=Unusual patterns && volume=low)
  Then
  Storage Repository=Blockchain based Electronic Medical Record
4. If (Patient= Famous Personality && health condition = Good)
  Then
  Storage Repository=Blockchain based Electronic Health Record
5. If (Patient= Famous Personality && health condition = Serious)
  Then
  Storage Repository=Blockchain based Electronic Medical Record
6. If (Data of type Disease)
  Then
  Store data in Disease Registry

**Algorithm 2:** Health repository recommendation system ().

Step 1: Begin
Step 2: data collected from various data sources;
Step 3: Call Association Mapping ();
Step 4: For each Health Data Block ϵ HB do
Step 5: Select the Supervised Machine learning algorithm;
Step 6: Train the Data block HB;
Step 7: Apply Heuristic Rule;
Step 8: If (Accuracy ≥ Threshold)
Step 9: Test data;
Step 10: Allocate the Health Data Block
HB → Health Repository HR;
Step 11: Send (Recommend Repository to Patients);
Step 12: Break;
Step 13: Else
Step 14: Continue;
Step 15: End; End; End;

## Results and Discussion

Research was conducted on supervised machine learning classification techniques. Using the WEKA tool, different classification algorithms were tested. The study used an Intel Core i7 6700H processor with up to 3.5 GHz and 16 GB of RAM. The dataset was divided into training and test sets. Data preprocessing is performed prior to analysis. To train the data in the recommended health repository, linear regression data blocks and user and health professional preference rules have been used. During this experiment, we determine whether the classifiers can learn how to classify data distributions. The training datasets each contain 400, 800, 1200, and 2000 instances. Table 3 shows the mapped sample training dataset.

**Table 3:** Mapped sample training data set.

**Information block**	**Sensitivity data**	**Volume**	**Context of medical care**	**Social status**	**Profile visibility**	**Patient status**	**Health repository**
Data Block 1	1	2	3	3	high	Typical	Blockchain based electronic health record
Data Block 2	2	5	3	5	Low	Typical	Cloud electronic health record
…….	…	…	…	….	…	….	…..
Data Block n	3	2	3	2	1	Abnormal	Electronic medical record

Four different classifiers were run on four datasets to test whether a machine learning algorithm could choose an appropriate storage medium, NaïveBayesSimple, Multilayer Perceptron, Random Forest Classifier, Random Tree and the IB1 algorithm are four different types of classifiers trained here. Several classification techniques were compared using Python to determine their accuracy scores (32).

### Classification Model Accuracy

1. Confusion matrix.
2. Classification measure.

#### Confusion Matrix

In the confusion matrix, N is the number of target classes, and N is the number of rows. It is used to evaluate the performance of a classification model. Machine learning is used to predict target values from the actual values in the matrix. True Positive (TP) and True Negative (TN) rates should be high and False Positive (FP) and False Negative (FN) rates are low for a successful model. A confusion matrix as is always more appropriate as a machine learning model evaluation criterion when working with an imbalanced dataset.

#### Classification Measure

As an evaluation measure, the classification measure is used in addition to the confusion matrix. They are:

1. Accuracy.
  (5) $$\begin{array}{l} \text{Accuracy}\;=\;\frac{\text{TP}+\text{TN}}{\text{TP}+\text{TN}+\text{FP}+\text{FN}}\;0.0<\text{Accuracy}<1.0 \end{array}$$
2. Precision.
  (6) $$\begin{array}{l} \text{Precision}\;=\;\frac{\text{TP}}{\text{TP}+\text{FP}} \end{array}$$
3. Recall.
  (7) $$\begin{array}{l} \text{Recall}\;=\;\frac{\text{TP}}{\text{TP}+\text{FN}} \end{array}$$
4. F1-Score.
  (8) $$\begin{array}{l} \text{F1-Scrore}\;=\;2\frac{\text{Recall}*\text{Precision}}{\text{Recall}+\text{ Precision}} \end{array}$$
5. Sensitivity and specificity.
  (9) $$\begin{array}{l} \text{Sensitivity}=\frac{\text{TP}}{\text{TP}+\text{FN}} \end{array}$$
  (10) $$\begin{array}{l} \text{Specificity}=\frac{\text{TN}}{\text{TN}+\text{FP}} \end{array}$$
6. Root mean square error.

Modified Mean Square Error (MSE) is a variation of Root Mean Square Error (RMSE). Measuring the mean square error squared is equivalent to this metric. The RMSE of an ideal model is zero, just as the MSE and MAE are zero.


(11) $$\begin{array}{l} \text{RMSE}=\sqrt{\frac{1}{\text{n}}\sum_{\text{i}=1}^{\text{n}}{\left(\text{Actual Values}-\text{Predicted Values}\right)}^{2}} \end{array}$$

### Result Analysis

**Figure 4:**
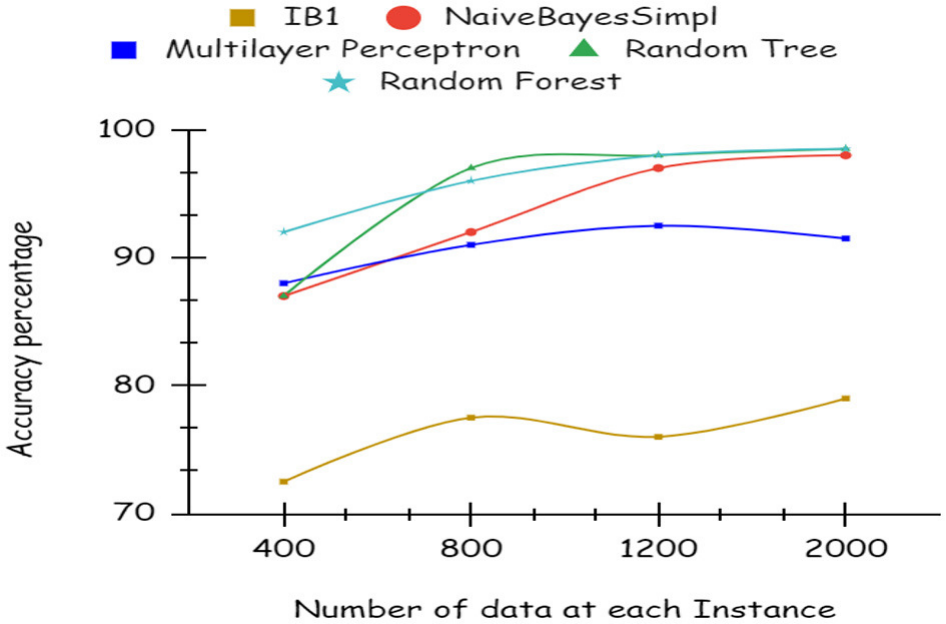
Accuracy using 10-fold cross validation.

**Figure 5:**
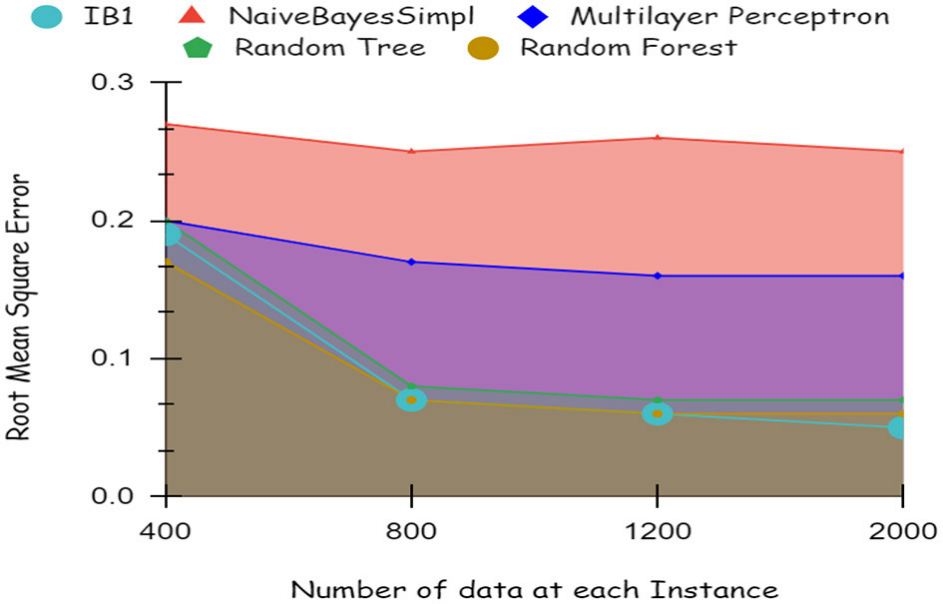
RMSE using 10-fold cross validation.

**Figure 6:**
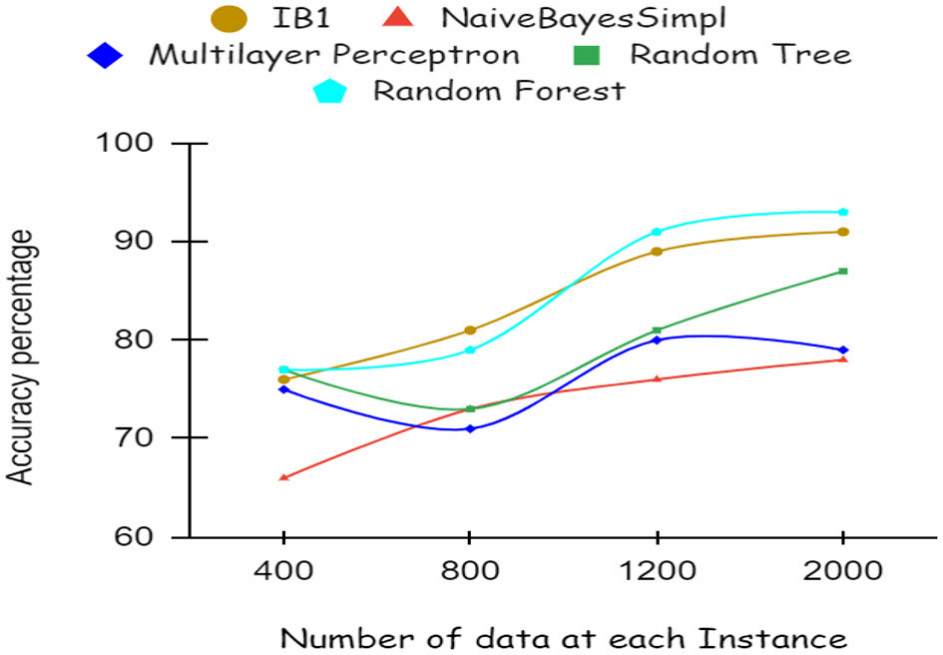
Accuracy of percentage split dataset.

As illustrated by the graph in Figure 4, Random Forest classifiers become more accurate as the number of instances increases, as shown by a 10-fold cross-validation analysis. A balanced ratio of each class was found in the dataset of 1,200 records, thus all classifiers performed better. The Random Forest performed best, with 98.21% accuracy. On the 2,000-record dataset, however, all classifiers had lower accuracy, largely because the dataset was skewed. Compared to other classifiers, Random Forest exhibits lower root mean square error in Figure 5. Figure 6 illustrates the percentage split results, which are less accurate than the cross-validation results presented in 10-fold cross-validation.

**Figure 7:**
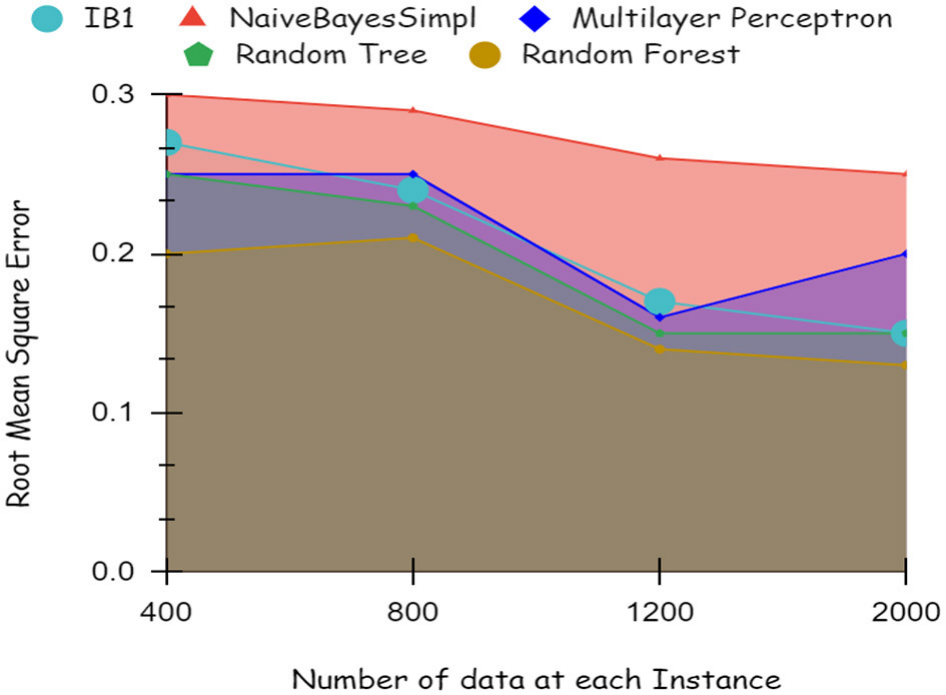
RMSE of percentage split dataset.

By using a percentage split, 80% of the data were used for training and 20% for testing. The classifier is trained only once, as seen in Figure 7, which demonstrates low accuracy and large RMSE. Artificial intelligence is a technique for deep learning.

**Figure 8:**
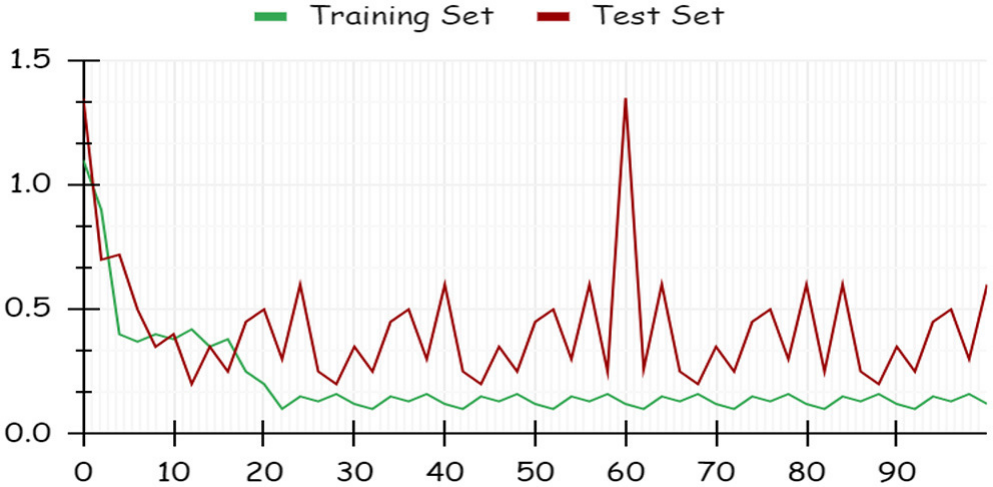
Performance loss of training and test set.

**Figure 9:**
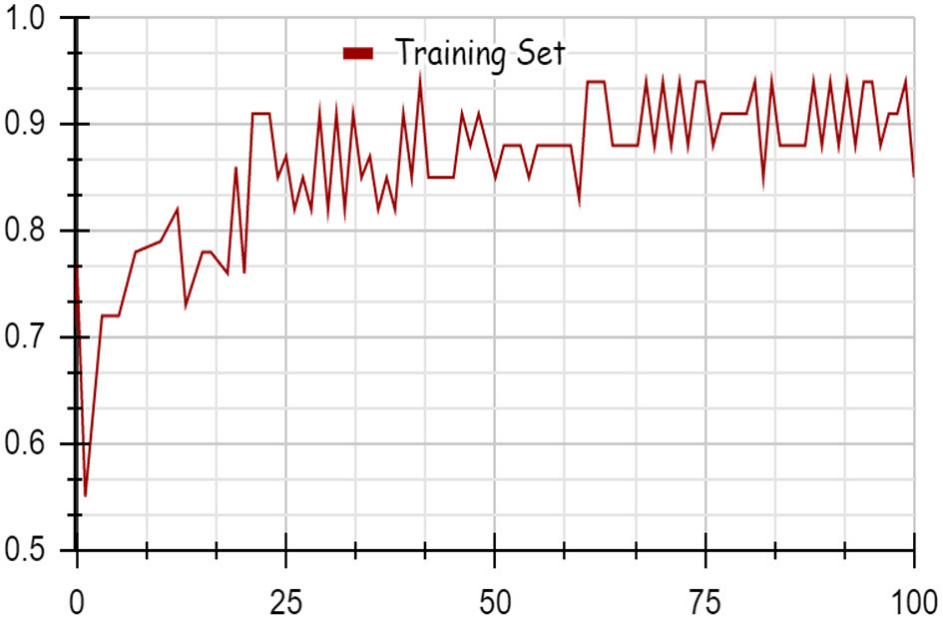
Performance accuracy of training and test set.

**Figure 10:**
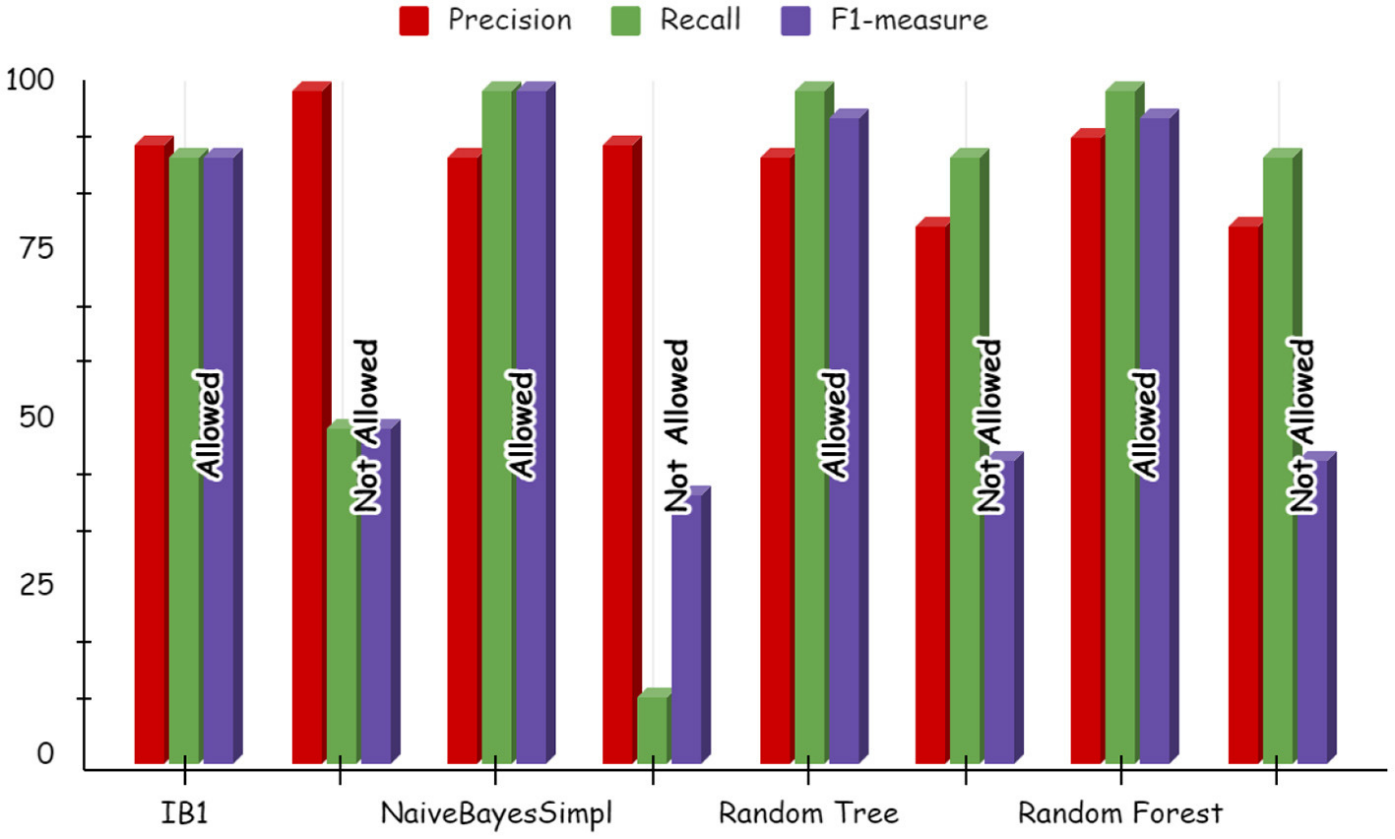
Deep learning results for cloud electronic health record.

**Figure 11:**
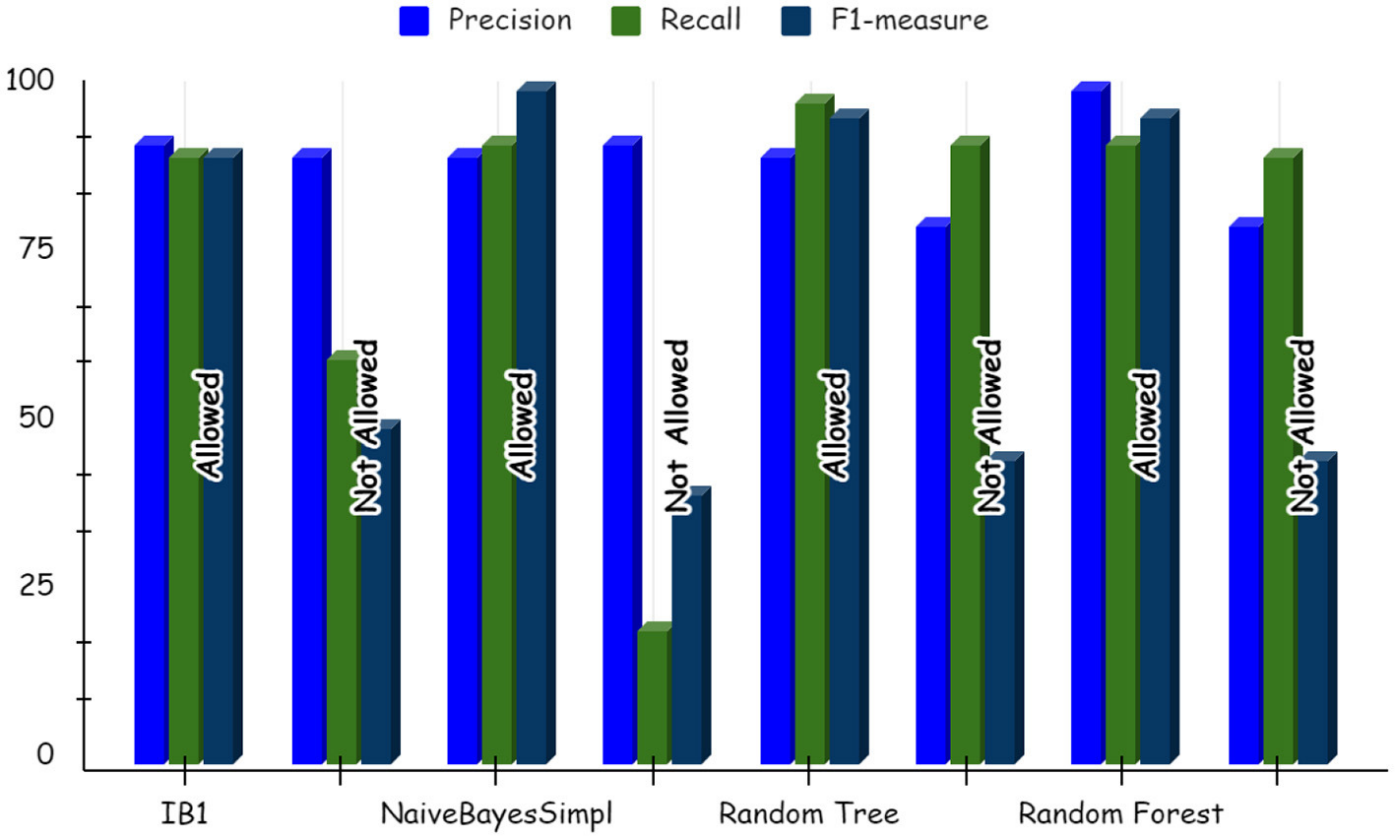
Deep learning results for blockchain based electronic health record.

**Figure 12:**
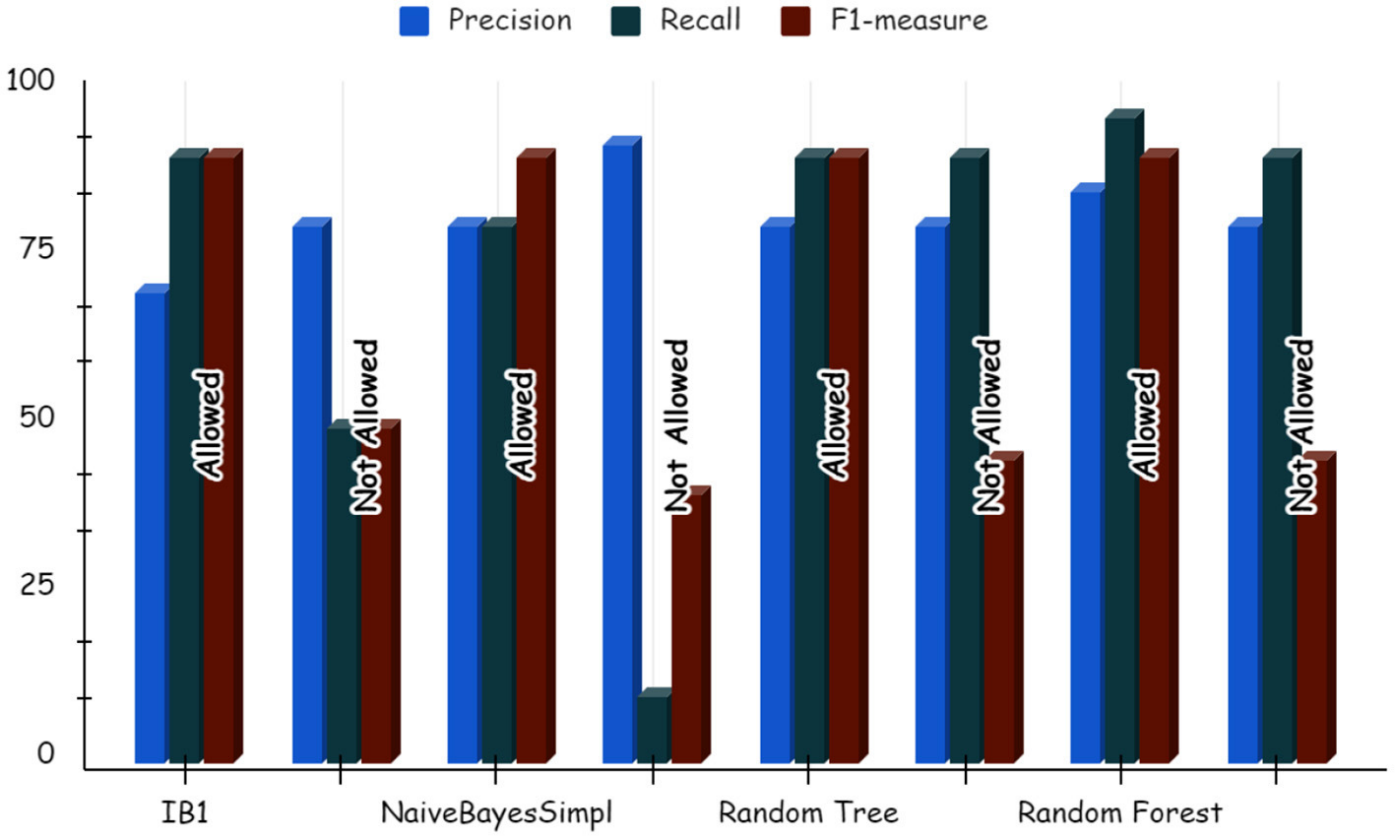
Deep learning results for electronic medical record.

Using deep learning networks, unstructured or unlabeled data can be learned unsupervised. Real-world health repositories are usually recommended based on unstructured and unlabeled datasets. For our synthetic dataset, we analyzed the accuracy using a deep learning algorithm. A deep learning model is run on the synthetic dataset, and it shows 88.70 percent accuracy. It is implemented in Python. There are three hidden layers in the model; the first of these layers has 100 output nodes, while the second and third have five output nodes each. Training is done with 100 iterations and eight batches are used. The training dataset is shown in Figures 8, 9, with a Y-axis showing the loss and X-axis showing the number of iterations. A deep learning classifier and a machine learning classifier are displayed in Figures 10–12 for the classification. With reference to recall, F1-measure, and precision, the Random Forest classifier outperformed the other tested classifiers. Classes that were allowed and those that were not were included in the experiment. In terms of recall, precision, and F1-measure, the Random classifier scored 93, 100, and 96% for cloud electronic health records, 100, 92, and 96 for blockchain-based electronic health records, and 85, 96, and 90 for electronic medical records. In terms of the allowed class, the rest of the experimented models perform well. In terms of the disallowed class, they did not perform well.

The accuracy of the classifier supports the use of machine learning to map the health storage mediums to health data blocks. Given the growing volume of health data that will need to be stored and accessed globally, this machine learning model may play a crucial role in improving storage and access arrangements in the future. This will make health data storage easy and straightforward for consumers. In addition, they would be able to ensure that the size of the data store is manageable. It can help to determine which storage solution best fits the requirements of different data assets using a machine learning model.

### Mapping of Health Data Parameters to Repositories

Medical technology is expected to develop health record systems in the future. Health records are taking on novel forms as a result of the expansion of medical data. As described below, the proposed system will support various data variations and health records. First, the system requests the ratings for the latest health record on the basis of health parameters from the IT staff and healthcare professionals. Second, the system relabels instances from the entire training dataset. As soon as a new instance is created, the old instances' labels do not change.

## Conclusion

Health data will increasingly be preserved in a variety of repositories, so patients can select the repository that best meets their needs. Patients are realistically expected to avoid using a single repository for all their health data because the context of treatment, patterns of data, and legal constraints may change. To automate the storage decision, a selection algorithm must be developed. This is especially relevant in the case of constantly streaming health data. The process of choosing the right repository is complicated. In addition to knowledge of storage features used for interoperability, data security, and privacy, regulatory concerns must also be considered. To preserve confidentiality, we propose distributing health data among various vendors. By keeping medical records together, confidentiality will also be preserved. Based on factors like data type, sensitivity level, significance, patient safety, and privacy requirements, this model can recommend which health data blocks should be stored on which storage medium. When applied to the dataset generated, random forest yielded the highest accuracy of 96.4%. Accuracy of algorithms depends on the dimension, origin, and nature of the data. As a result, we intend to evaluate these various algorithms with different characteristic datasets in the near future. In the future, we will implement a role-based access control system to store medical record information by integrating the health repository recommendation system to allow access to the health records based on the permission of patients.

## Data Availability Statement

The original contributions presented in the study are included in the article/supplementary material, further inquiries can be directed to the corresponding author/s.

## Author Contributions

VM and CK: conceptualization, methodology, investigation, data curation, and writing—original draft preparation. SB, AM, and PH: software, validation and visualization, and resources. VM, AM, and PH: formal analysis. VM, SB, and AM: writing—review and editing and supervision. VM, CK, SB, AM, and PH: project administration. All authors have read and agreed to the published version of the manuscript.

## Conflict of Interest

The authors declare that the research was conducted in the absence of any commercial or financial relationships that could be construed as a potential conflict of interest.

## Publisher's Note

All claims expressed in this article are solely those of the authors and do not necessarily represent those of their affiliated organizations, or those of the publisher, the editors and the reviewers. Any product that may be evaluated in this article, or claim that may be made by its manufacturer, is not guaranteed or endorsed by the publisher.
